# Miscellaneous

**Published:** 1889-10

**Authors:** 


### MISCELLANEOUS.

A Cure for Alcoholism.—One who has had much experience in reformatory institutions for drunkards has lately given his testimony as to the curability of the habit. He stoutly insists that alcoholism is not a disease, and that it is possible for every man, no matter how long he has been a drunkard, to recover from the habit. Reformation requires a cultivation of the moral sense, a strengthening of the will, a cutting off of all evil associations and all bad habits, and total abstinence from the use of liquor. It will never do for a drunkard who is trying to reform to stop short of absolute denial of alcohol; it is simply a delusion for one like him to think he can limit himself to moderation. It is the opinion of the expert in question that the most marked results of inebriety are not physical, but mental and moral. They do not produce disease to the extent generally believed.

Disinfection by Steam.—The recent researches of Esmarch seem to indicate that the destruction of bacteris by steam does not depend so much on the temperature as upon the degree of saturation of the steam. If there is much air with it, the power of destroying organic germs is very much diminished. In the course of some experiments on the spores of the anthrax bacillus it was found that while superheated steam which was not in a condition of saturation at a temperature of 20 degrees centigrade was unable to destroy the spores in half an hour. satuated steam at 100 degrees destroyed them in from five to ten minutes. This information will have to be borne in mind in the construction of apparatus for disinfecting by means of steam, the uncertainty of which is thus explained in a way which will enable it to be remedied in the future.

The Sugar of Milk.—The manufacture of lactine, or sugar of milk, is a new industry in this country which promises to attain importance in the milk-producing districts. Heretofore the manufacture has been confined to Switzerland and Bavaria. The wholesale price is twenty-five to thirty cents a pound. The first plant for the manufacture of lactine in this country was established a year or two ago at Hamburg, N. J. Another factory has recently been started at Oxford, N. Y. A third factory is about to be opened at Unionville N. Y., for the manufacture of the article on a large scale by a new process, which is claimed to yield a superior product at a reduced cost of production.

Imitations of Old Bronze.—An excellent imitation of old bronze has been introduced in some of the art products of that character. It is well known that the repeated applications to copper or brass of alternate washes of dilute acetic acid and exposure to the fumes of ammonia result in a very antique looking and highly prized antique green bronze ; but a more rapid method of producing this beautiful appearance has long been a desideratum. It is now found that this may be accomplished by immersing the articles in a solution of one part perchloriate of iron in two parts of water, the tone acquiring darkness with length of immersion, or the materials may be boiled in a solution of nitrate of copper. It is also found practicable to insure the desired effect by immersing the articles in a solution of two ounces of nitrate of iron and the same quantity of hyposulphite of soda in half a pint of water, drying and burnishing completing the process.

DEATH FROM TIGHT LACING.—A verdict of death from tight lacing is, perhaps, still to be sought among the curiosities of law. But a Birmingham jury have come near it in a verdict of death from pressure round the waist. The victim was a poor servant girl who died after a fright, and her death was attributed by medical witnesses to the fact that she was too tightly belted to enable her to stand the wear and tear of any sudden emotion. She was a notorious tight lacer; her collar fitted so closely that it was impossible to loosen it at the critical moment, and under her stays she wore a belt so remorselessly buckled as to prevent the free circulation of the blood.—*St James' Gazette.*

NEW YORK'S POPULATION.—The population of the City of New York, by the latest and closest calculation, is 1,753,610 souls. The valuation of the real and personal estate is $1,553,442,435.66, as follows: Real estate, $1,302,818,879; personal estate, $250,623,556.66. The bonded debt in 1888 was $132,445,095, and the sinking fund commission holds in bonds and stocks $44,324,690, leaving a net debt of $88,120,405, the interest on the whole being $7,140,254, at the rate of from 7 to 2 per cent.—*New York Evening Telegram.*

A CURE FOR DRUNKENNESS.—A half ounce of ground quassia steeped in a pint of vinegar, is recommended highly as a cure for drunkenness. A teaspoonful in a little water should be taken every time the liquor thirst is felt. It satisfies the cravings and produces a feeling of stimulation and strength.

FRECKLES.—These evidences of sunshine may be removed with an ointment composed of white precipitate and subnitrate of bismuth, of each, one drachm; glycerine ointment, ½ ounce. Each freckle is touched with the ointment every other day.

